# Comparative Characteristics of Immunochromatographic Test Systems for Tylosin Antibiotic in Meat Products

**DOI:** 10.3390/s24216865

**Published:** 2024-10-25

**Authors:** Lyubov V. Barshevskaya, Dmitriy V. Sotnikov, Elena A. Zvereva, Boris B. Dzantiev, Anatoly V. Zherdev

**Affiliations:** A.N. Bach Institute of Biochemistry, Research Center of Biotechnology of the Russian Academy of Sciences, Leninsky Prospect 33, 119071 Moscow, Russia; lyubov.barshevskaya@yandex.ru (L.V.B.); sotnikov-d-i@mail.ru (D.V.S.); zverevaea@yandex.ru (E.A.Z.); zherdev@inbi.ras.ru (A.V.Z.)

**Keywords:** lateral flow assay, immobilized antibodies, veterinary drugs, livestock, gold nanoparticles

## Abstract

Tylosin (TYL) is a macrolide antibiotic widely used in animal husbandry. Due to associated health risks, there is a demand for sensitive methods for mass screening of TYL in products of animal origin. This article describes the development of lateral flow immunoassays (LFIAs) for TYL detection using direct (anti-TYL antibodies conjugated with nanoparticles) and indirect antibody labeling (anti-species antibodies conjugated with nanoparticles and combined with native anti-TYL antibodies). The choice of LFIA conditions, such as concentrations of hapten–protein conjugates, specific antibodies, and gold nanoparticle (GNP) conjugates with antibodies, as well as incubation time of reagents and the concentration of detergent in the sample buffer, is presented. The achieved limits of TYL detection using LFIAs with indirect labeling were 0.8 ng/mL (visual) and 0.07 ng/mL (instrumental), compared to 4 ng/mL (visual) and 0.4 ng/mL (instrumental) for the case of direct labeling. The sensitivity of the LFIA using the indirect format was up to seven times higher, allowing the determination of the target analyte at low concentrations. TYL detection in ground meat using LFIA with indirect antibody labeling ranged from 76–119%.

## 1. Introduction

The widespread use of antibiotics in various areas of agriculture continues to raise great concerns due to numerous negative consequences [1,2]. Animal husbandry now consumes 73% of worldwide antibiotics sold, significantly outpacing their medical use [3]. Moreover, antibiotics are very often applied by livestock breeders as preventive measures, not for the treatment of diagnosed diseases. Such excessive use of antibiotics, because of their various negative effects, is harmful not only to humans as consumers of animal products but also to the environment [4,5,6].

One of the main problems related to antibiotics is antibiotic resistance, which poses a serious threat to humans [7,8]. The consumption of food products contaminated with antibiotics leads to a number of health risks, including direct negative consequences (e.g., allergic reactions, microflora disruption, etc.) and a decrease in the number of antibiotics that can be effectively used to treat infectious diseases [9]. In addition, antibiotics contaminate soil and water [10,11,12], which also contributes to the antibiotic resistance.

Tylosin (TYL) is one of the most commonly used antibiotics from the macrolide family; it is applied for the treatment and prevention of infectious diseases, as well as a growth promoter [13,14]. Although TYL is approved for use in all types of livestock animals, its uncontrolled application leads to the growth of the problems listed above. Thus, sensitive methods for TYL detection in livestock products to monitor manufacturing chains and protect consumers are in high demand.

Traditional methods for TYL detection are chromatographic ones (e.g., LC-MS and HPLC) [15,16]. These methods are characterized by high sensitivity and specificity, but their implementation is possible only under laboratory conditions. Among immunochemical methods for TYL detection, enzyme-linked immunosorbent assay (ELISA) has widespread use [17,18]. ELISA is well characterized as sensitive assay, but it takes several hours and includes complex sample preparation.

Another perspective immunoanalytical method is the lateral flow immunoassay (LFIA). LFIA is a simple, rapid, and reproducible technique that does not require stationary equipment or specially trained personnel [19,20,21]. All this makes LFIA a promising tool for non-laboratory mass screening of livestock products for tylosin content. To date, several LFIAs have been developed for TYL detection in various livestock products (see [22,23] for examples). Nevertheless, the development of new approaches to increasing the sensitivity of LFIA for TYL continues [24,25].

**Figure 1:**
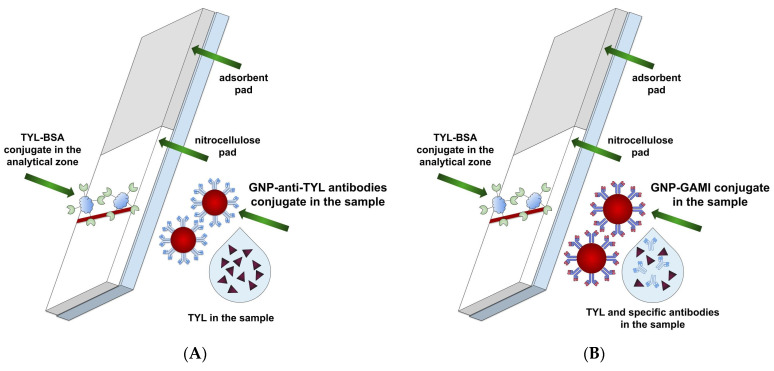
Scheme of LFIA with direct (**A**) and indirect (**B**) antibody labeling.

In this paper, for the first time, two LFIA approaches with direct and indirect antibody labeling (i.e., based on specific antibodies conjugated with nanoparticles or on the combination of native specific antibodies and anti-species antibodies conjugated with nanoparticles, respectively; see Figure 1) were comparatively characterized for tylosin detection in meat products. We studied how the parameters of LFIA, such as the concentration of reagents, incubation time, and flow rate, affect the detection limit and intensity of coloration of the test strips. The obtained data demonstrate the advantages and high competitive potential of the indirect labeling approach, state the optimal conditions for its application, and provide the possibility of more sensitive monitoring of tylosin.

## 2. Materials and Methods

### 2.1. Reagents and Materials

The following compounds were used in the study: goat anti-mouse (anti-species) antibodies (Imtek, Moscow, Russia), chloroauric acid, TYL, Tween-20, sodium citrate, sodium azide (Sigma-Aldrich, St. Louis, MO, USA), and bovine serum albumin (BSA) (Boval Biosolutions, Cleburne, TX, USA). Anti-TYL monoclonal antibodies and the TYL–BSA conjugate were from Creative Diagnostics (Shirley, NY, USA). All other salts, acids, and alkalis used in this study were of analytical or chemical grade. Deionized water cleansed by the Simplicity system (Millipore, Burlington, MA, USA) was used to prepare gold nanoparticles and their conjugates.

The working nitrocellulose membrane CNPC SS-12, AP-045 adsorption membrane, and L-P25 plastic support (all from Advanced Microdevices, Ambala Cantt, Haryana, India) formed the set of materials for test strip preparation.

### 2.2. Synthesis and Characterization of Gold Nanoparticles

Gold nanoparticles (GNPs) were synthesized according to the approach proposed by Frens [26] and based on the reduction of chloroauric acid by citrate anions. The detailed protocol adopted for obtaining nanoparticles with an average diameter about 25 nm was taken from [27]. 

To characterize GNPs by transmission electron microscopy (TEM), a CX-100 microscope (Jeol, Tokyo, Japan) was used, as described in [27], and the data were processed using the Image Tool program, version 3.0 (UTHSCSA, San Antonio, TX, USA). Dynamic light scattering (DLS) measurements of GNPs were implemented using the Zetasizer Nano ZSP nanoparticle analyzer and Zetasizer software, version 7.11 (both from Malvern Instruments, Malvern, UK).

### 2.3. Conjugation of GNPs with Antibodies

The GNPs were conjugated with anti-TYL antibodies or anti-species antibodies by adsorption immobilization at pH 8.5, as described in [27]. The obtained conjugates were separated from unbound proteins by centrifugation, redissolved in 10 mM Tris (pH 8.5) with 1% BSA and 1% sucrose, and stored with the addition of sodium azide (0.05%) at 4 °C.

### 2.4. Determination of the Amount of Antibodies Conjugated with GNPs

The composition of the obtained antibody–GNP conjugates was determined using the technique proposed in [28] and based on the registration of the fluorescence of tryptophan residues in the antibodies. Two solutions were characterized for this purpose: (i) the antibodies initially taken for the conjugation without adding stabilizers, and (ii) the non-conjugated antibodies (namely, supernatants of the reaction mixture after the conjugation process). To exclude the influence of supernatant compounds on the registered fluorescence, the antibody solutions were mixed with antibody-free supernatants, and their fluorescence was measured to calibrate the determination of antibody concentrations. The content of bound antibodies was calculated based on the difference between the initially taken and non-bound antibodies. The fluorescence spectra were recorded in Nunc MaxiSorp white microplates (Roskilde, Denmark) using a microplate reader (Perkin Elmer En Spire 2300, Waltham, MA, USA) with excitation and emission wavelengths of 280 nm and 350 nm, respectively. The details of the measurements and calculations are given in the Supplementary Information. 

### 2.5. Test Strips Production

An analytical zone was formed on a working nitrocellulose membrane using an IsoFlow automatic dispenser (Imagene Technology, Lebanon, NH, USA) by applying the TYL–BSA solution (0.5 mg/mL, consumption 1 μL/mm) in 50 mM potassium phosphate buffer, pH 7.4, with 0.1 M NaCl (PBS). The working membrane was then dried at room temperature for 24 h. A multimembrane composite (including the CNPC SS-12 working membrane, AP-045 adsorption membrane, and L-P25 plastic support) was assembled, cut into 3.5 mm-wide strips using an Index Cutter-1 automatic guillotine cutter (A-Point Technologies, Brea, CA, USA), and stored at room temperature in sealed packages containing silica gel.

### 2.6. Implementation of LFIA

#### 2.6.1. LFIA with Direct Labeling of Antibodies

To perform the analysis, TYL at concentrations of 20–0.0013 ng/mL was mixed with the GNP–anti-TYL antibody conjugate (OD_520_ = 0.07) in 20 µL PBS with 1% Tween-20 (PBST) in microplate wells. The test strips with immobilized TYL–BSA conjugates were introduced into the wells in a vertical position. After 5 min, the test strips were removed, placed on a horizontal surface, and immediately scanned with a Canon Lide 90 flatbed scanner (Canon, Tokyo, Japan) at a resolution of 600 dpi without contrast and color correction modes.

#### 2.6.2. LFIA with Indirect Labeling of Antibodies

The TYL in concentrations varied from 20 to 0.0013 ng/mL (or spiked meat extract) were mixed with the GNP–goat anti-mouse antibody conjugate (GNP-GAMI, OD_520_ = 1.0) and anti-TYL monoclonal antibodies (25 ng/mL) in 20 µL of PBST in microplate wells and left to incubate for 5 min. After that, the introduction of test strips and the following steps were carried out as described above.

#### 2.6.3. LFIA with Indirect Labeling of Antibodies and Step-by-Step Addition of Reagents

The TYL concentrations varied from 20 to 0.0013 ng/mL (or spiked meat extract) were mixed with anti-TYL monoclonal antibodies (25 ng/mL) in 20 µL of PBST. After 5 min, the test strips were removed and placed in microplate wells with 20 µL of the GNP-GAMI conjugate (OD_520_ = 1.0) in PBST. After 5 min of incubation, they were finally removed and scanned as described above.

### 2.7. Meat Samples Preparation

Samples were prepared in accordance with [29]. Ground beef free from TYL was purchased at a local grocery store. To prepare the extract, 3 g of the ground meat was mixed with 6 mL of ethyl acetate and incubated for 5 min. The resulting mixture was then centrifuged at 2000 *g* for 5 min at 4 °C, after which the supernatant (4 mL) was collected, evaporated at 60 °C, redissolved in 1 mL of PBST, and used for the analyses.

### 2.8. Processing Test Strip Images and Calculating Assay Parameters

The images obtained from the scanning were analyzed using the Total Lab v2009 software (Nonlinear Dynamics, Newcastle upon Tyne, UK), as described in [27]. The registered concentration dependencies of the analytical zone’s coloration were approximated by a 4-parameter sigmoid function.

The instrumental limit of TYL detection was considered to be the concentration point that corresponds to a 10% decrease in the analytical line’s coloration. The TYL concentration, causing a visually observable disappearance of coloration of the analytical zone, was stated as the visual detection limit.

## 3. Results

### 3.1. Characterization of GNP

GNPs were synthesized under conditions that were chosen based on recommendations for the preferable use of GNPs with diameters in the range 20–40 nm for LFIA [30,31]. The obtained nanoparticles and their conjugates with antibodies were characterized using three methods: TEM, DLS, and spectrophotometry.

**Figure 2:**
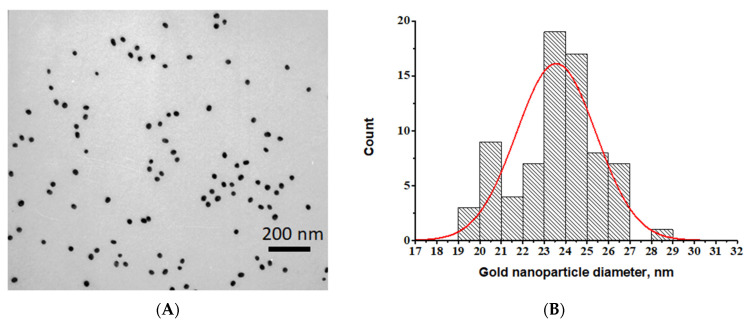
Transmission electron microscopy data for GNPs. (**A**) Micrograph. (**B**) Histogram of diameters distribution.

According to the TEM data, the average diameter of the obtained nanoparticles was 24.51 ± 3.02 nm (n = 300, the minimum value was 12.34 nm, and the maximum value was 26.58 nm) with a degree of ellipticity of 1.13 ± 0.05 (Figure 2).

**Figure 3:**
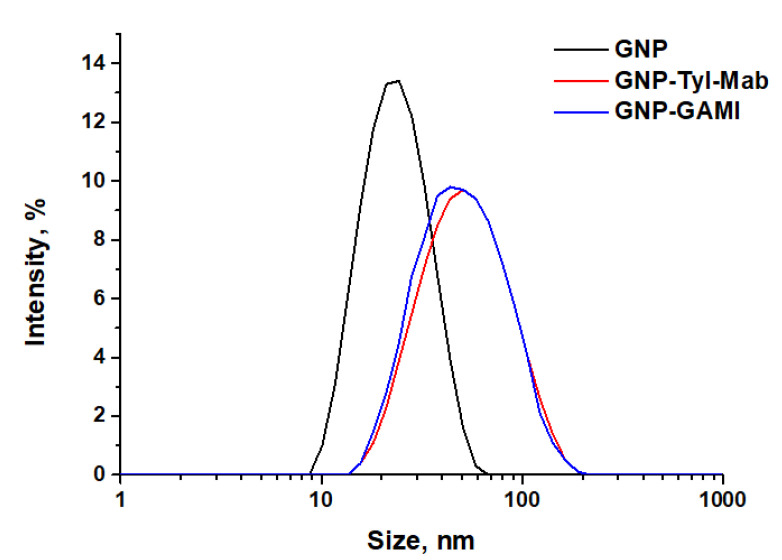
Distributions of diameters for GNPs and their conjugates with antibodies based on DLS measurements.

The synthesized GNP and the obtained GNP conjugates with antibodies were also characterized by DLS. DLS allows for determining the hydrodynamic diameter of a particle in a solution, taking into account the formation of hydrate shells around the particle. Figure 3 shows the size distribution of the GNPs. The hydrodynamic diameter of the initial GNPs was 30.7 ± 6.8 nm. The difference in nanoparticle diameter obtained by the DLS method compared to the TEM method was due to the contribution of the hydration shell of the particle, which was accounted for by the DLS.

The DLS diameters of GNP–GAMI and GNP–anti-TYL antibodies were 50.8 ± 5.6 and 57.8 ± 3.9 nm, respectively. The increase in diameters (compared to 30.7 ± 6.8 nm for the initial GNPs) resulted from the adsorption of the antibodies on the GNP surface and is in good accordance with size of immunoglobulin molecules.

**Figure 4:**
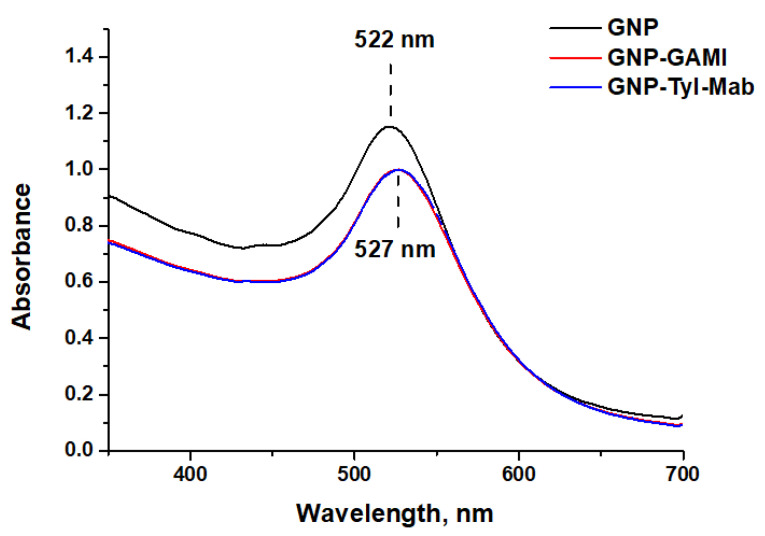
UV-vis absorption spectra of GNPs and their conjugates with antibodies.

The UV-vis absorption spectra of the synthesized GNP preparations are shown in Figure 4. The absorption maximum was localized in the region of 522 nm. The position of the maximum, the width, and the absence of additional peaks in the spectrum indicate the homogeneity of the GNPs. A shift in the peak position of the spectra to 527 nm indicates successful conjugation of GNPs with antibodies [32]. The local dielectric environment of GNPs changes as a result of antibodies binding to their surface. This causes a shift in the local plasmon resonance peak that can be registered by absorption spectrophotometry [33,34].

### 3.2. Determination of the Amount of Antibodies in Conjugates with GNPs

We estimated the total surface area of the GNPs and the maximal possible coverage of this surface by immobilized antibodies (immunoglobulins G, IgG) as previously proposed in [35] (see detailed calculations in the Supplementary Information). Based on the TEM data about the average diameter of the GNPs, the maximal amount of antibodies for monolayer coating of the surface of one nanoparticle was 96. We used some excess of antibodies for the conjugation (10 μg/mL), providing an IgG:GNP ratio in the reaction mixture of 120:1.

To measure the degree of IgG coupling, we applied a technique based on specially adopted measurements of tryptophan fluorescence for added and non-bound antibodies (see Section 2.6). To exclude the risk of random deviations, antibodies were added to supernatants after GNP centrifugation at three final concentrations of 10, 20, and 30 μg/mL, and changes in the solution volumes were included in the calculations. The observed degrees of antibody coupling (see Supplementary Information) were highly reproducible: 88–89% for anti-TYL antibodies and 80–82% for anti-species antibodies. The IgG:GMP ratio varied between 96:1 and 107:1, which is close to the theoretical limit for monolayer binding. Note that the DLS data also indicated a diameter increase of ~20 nm (two lengths of IgG molecules) for IgG–GNP conjugates compared with the initial GNPs. Therefore, the chosen conjugation conditions provided the highest monolayer coverage of GNPs without the formation of less stable additional layers [35].

### 3.3. Choice of Conditions for Tylosin LFIA with Direct and Indirect Labeling of Specific Antibodies

#### 3.3.1. LFIA with Direct Antibody Labeling

**Figure 5:**
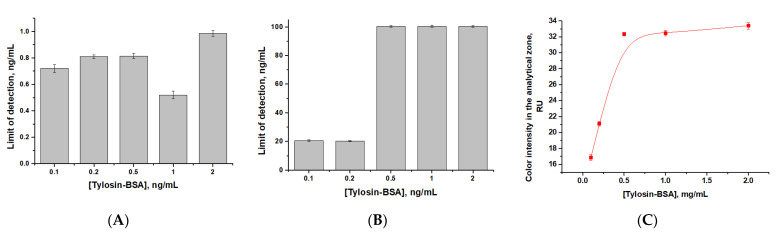
Dependencies of instrumental (**A**) and visual (**B**) detection limits, and intensity of coloration of the analytical zone (**C**) on the applied concentration of the Tyl–BSA conjugate.

Conditions for tylosin LFIA with direct antibody labeling were optimized. First, the concentration of the Tyl–BSA conjugate for immobilization in the analytical zone of the working membrane of the test strip was chosen. For this purpose, the dependence of the detection limit and the intensity of coloration of the analytical zone on the concentration of the adsorbed conjugate was studied. According to the data obtained (Figure 5), with varying the concentration of Tyl–BSA from 2.0 to 0.1 mg/mL, a decrease in the visual (100–20 ng/mL) and instrumental detection limits (1–0.7 ng/mL) was observed, as well as a decrease in the intensity of coloration of the analytical zone. The use of the lowest concentration of the conjugate (0.1 mg/mL) did not interfere with the visual assessment of the assay results. Therefore, for further work, the use of test strips with immobilized Tyl–BSA at a concentration of 0.1 mg/mL was chosen.

**Figure 6:**
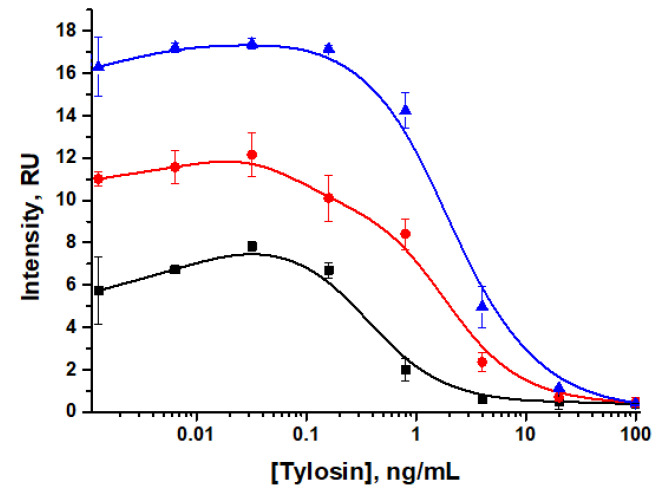
Concentration dependences of tylosin LFIA with direct antibody labeling for various concentrations of GNP–anti-TYL antibody conjugates (black curve—OD_520_ = 0.05; red curve—OD_520_ = 0.07; blue curve—OD_520_ = 0.1).

In the next stage, the concentration of the GNP–anti-TYL antibodies conjugate was varied. According to the data obtained (Figure 6), as this concentration decreased from OD_520_ = 0.1 to OD_520_ = 0.05, both visual (20–4 ng/mL) and instrumental (0.7–0.2 ng/mL) detection limits decreased. However, using the conjugate at the lowest concentration (OD_520_ = 0.05) made visual assessment of the analysis results difficult. Therefore, for further work, a concentration of the conjugate corresponding to OD_520_ = 0.07 was chosen.

Taking into account the selected parameters, TYL detection in PBST was carried out by varying the incubation time of the reagents (antigen and GNP-anti-TYL antibodies conjugate) from 0 to 5 min. As can be seen from Table 1, the visual LOD was 4 ng/mL, and the instrumental LOD varied in the range of 0.3–0.9 ng/mL. We chose to carry out the analysis without incubating the reagents, since this option provided the lowest instrumental LOD and quite an intense coloration of the analytical zone.

To analyze the changing time of reagent interaction during the liquid movement along the test strip, we considered the detergent Tween-20 as a known compound influencing the lateral flow processes in test strips [36]. The detergent was added to PBST at different concentrations. According to the results (Table 2), as the concentration of Tween-20 decreased (2–0.2%), the total analysis time varied from 5 to 10 min. Thus, the flow rate became slower with lower concentrations of detergent.

**Figure 7:**
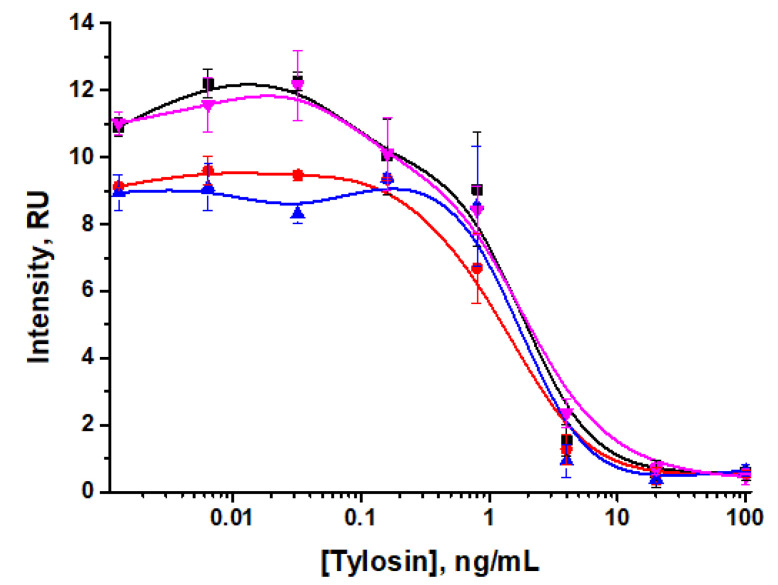
Concentration dependencies of tylosin LFIA with direct antibody labeling for various concentrations of detergent Tween-20 in PBST (black curve—2%; pink curve—1%; red curve—0.5%; blue curve—0.2%).

Another LFIA characteristic, the LOD, was determined to be 4 ng/mL (visual) and 0.4–1 ng/mL (instrumental), while the intensity of the analytical zone coloration was lower when using lower concentrations of Tween-20 (Figure 7), which also showed the impact of the time of reagent interaction. For intense coloration of the test strips, PBST with 1% Tween-20 was chosen. This solution provided a reliable visual assessment of the results within the shortest analysis time compared to lower detergent concentrations (0.2% and 0.5%) but it resulted in a longer duration of analyte detection.

#### 3.3.2. LFIA with Indirect Antibody Labeling

For LFIA with indirect antibody labeling, the dependencies of analytical zone coloration intensity on the TYL–BSA conjugate concentration, the concentration of specific antibodies, and the concentration of the GNP–GAMI conjugate were studied. As can be seen in Figure 7, the coloration intensity decreases with lower concentrations of TYL–BSA (1–0.2 mg/mL) and specific antibodies (100–6.25 ng/mL). The choice of optimal parameters for the tylosin test system was based on maintaining acceptable coloration intensity for the visual assessment of the analysis results while lowering the detection limit.

**Figure 8:**
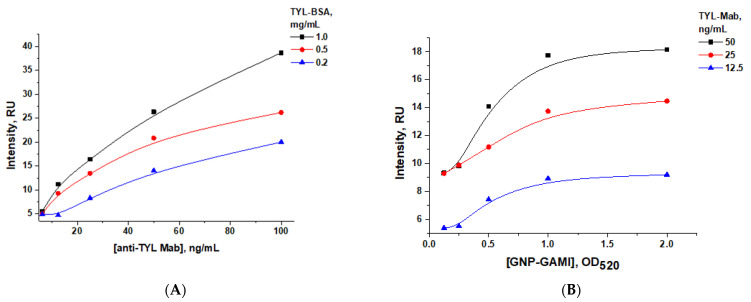
Dependencies of color intensity of the analytical zone for tylosin LFIA with indirect antibody labeling on TYL–BSA and anti-TYL antibody concentration (**A**), and the GNP–GAMI conjugate (**B**).

We chose a TYL–BSA concentration of 0.5 mg/mL and three concentrations of anti-TYL antibodies (50, 25, and 12.5 ng/mL) (Figure 8A), which were used to study the dependence of the coloration intensity of the analytical zone on the third parameter—the concentration of GNP–GAMI conjugate (Figure 8B). It was determined that using a marker conjugate at a concentration corresponding to OD_520_ = 1.0 and a specific antibody concentration of 25 ng/mL would provide an acceptable visual assessment of the analysis results without exceeding the LOD values (Figure 8B).

**Figure 9:**
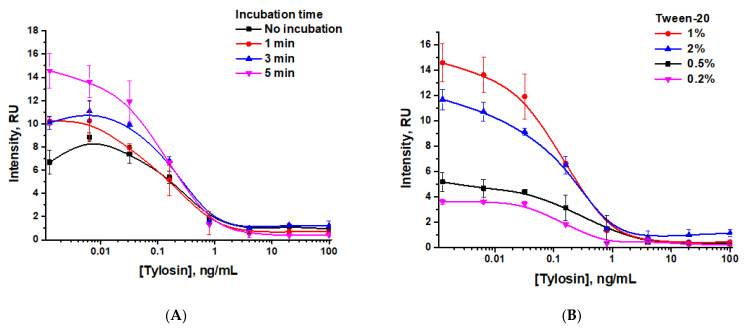
Concentration dependencies of tylosin LFIA with indirect antibody labeling for various incubation times (**A**) and detergent concentrations (Tween-20) in PBST (**B**).

After this, we studied the dependence of the LOD and color intensity in the analytical zone on the time of incubation and Tween-20 concentrations. As can be seen in Figure 9A and Table 3, when varying the incubation time of the reagents (the antigen, anti-TYL antibodies, and the GNP–GAMI conjugate) from 0 to 5 min, the visual LOD was 0.8 ng/mL, and the instrumental LOD ranged from 0.01–0.08 ng/mL, while the color intensity increased with longer incubation times (i.e., for more prolonged interaction of the reagents).

When varying the Tween-20 concentration in PBST to control the flow rate and time of reagent binding, the obtained LODs were 0.8 ng/mL for visual detection and 0.03–0.07 ng/mL for instrumental detection, while the intensity of coloration in the analytical zone was lower when using lower concentrations of Tween-20 (Figure 9B, Table 4). To provide acceptable coloration of the test strips, PBST with a Tween-20 concentration of 1% was chosen.

**Figure 10:**
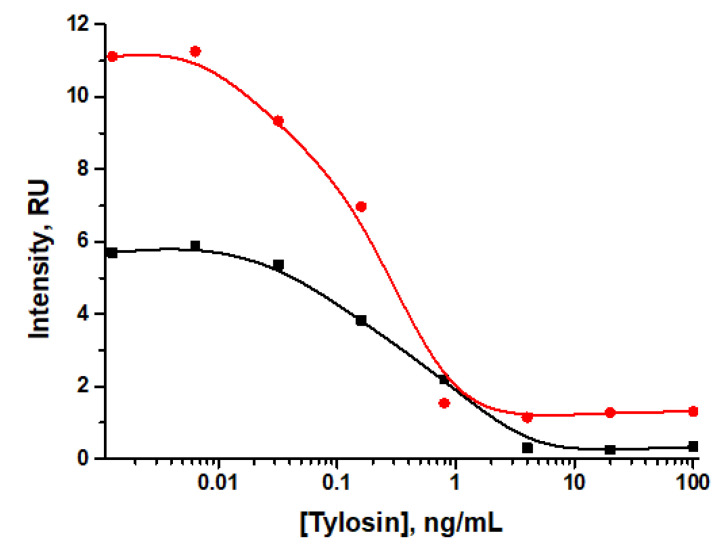
Dependence of LOD on preincubation (red curve) and step-by-step addition of reagents (black curve).

Taking into account the selected parameters, preincubation of reagents in LFIA with direct labeling was estimated. According to the data obtained (Figure 10), instrumental LOD corresponded to 0.04 ng/mL and 0.03 ng/mL for analysis with preincubation and step-by-step addition of reagents respectively, while visual LOD was 0.8 ng/mL (with preincubation) and 4 ng/mL (with step-by-step addition).

The intensity of coloration of the analytical zone was lower when performing the analysis without preincubation of reagents, which may subsequently lead to incorrect interpretation of the analysis results. Thus, the assay protocol with preincubation was selected for further work.

### 3.4. Assessment of Analytical Characteristics of LFIAs with Direct and Indirect Antibody Labeling

To select the optimal conditions for the analysis and the LFIA format that allows for detecting the target analyte at low concentrations, a comparative analysis of the parameters of test systems with direct and indirect antibody labeling was performed (Table 5). It was found that the lowest detection limit (both visual and instrumental) was obtained for LFIA with indirect antibody labeling and preincubation of reagents (5 min), and the analysis time in all three formats varied from 5 to 15 min. Based on the obtained data, the LFIA format with indirect antibody labeling was selected, where the reagent incubation time was 5 min, and a 1% Tween-20 concentration in PBST was used as a working buffer. Even though testing in this LFIA format took 15 min, which was the longest duration for the analysis, the achieved analytical parameters provided accurate TYL detection at low concentrations.

### 3.5. Approbation of LFIA with Indirect Antibody Labeling in Meat Extract

**Figure 11:**
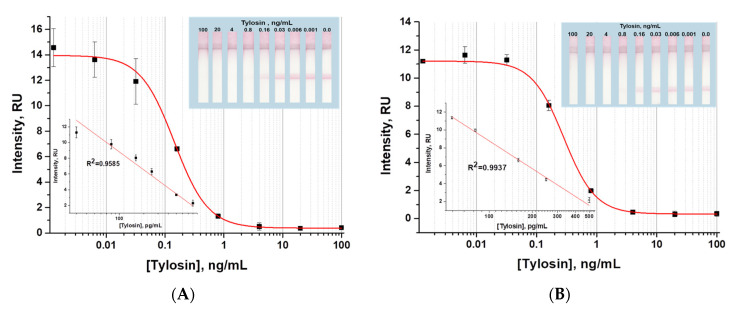
Calibration curves of indirect LFIA for tylosin in PBST (**A**) and in meat extract (**B**).

The indirect LFIA for TYL, identified as the most sensitive assay format, was tested with PBST and meat extract containing TYL at concentrations ranging from 100 to 0.001 ng/mL (Figure 11). The obtained visual and instrumental detection limits were 0.8 ng/mL (1.6 µg/kg) and 0.07 ng/mL (0.14 µg/kg), respectively, which were lower than the established maximum permissible levels for TYL content in foodstuffs of animal origin (100 µg/kg) [37].

To evaluate the developed LFIA for tylosin detection, meat samples were spiked with TYL. As shown in Table 6, the recovery values of TYL from the spiked samples varied between 76 and 119%. Thus, we can conclude that the proposed indirect LFIA corresponds to a high accuracy of TYL detection in products of animal origin.

### 3.6. Selectivity of Indirect LFIA for Tylosin

To test the selectivity of the developed test system, five antibiotics (tetracycline, chloramphenicol, neomycin, lincomycin, and cefapirin) were studied. After incubation (5 min), a test strip was immersed in the sample.

In addition, the effect of non-target analytes in the sample on specific binding was investigated. For this purpose, one of the five antibiotics mentioned above (300 ng/mL) was added to the test sample with tylosin (100 ng/mL), specific antibodies, and the GNP–GAMI conjugate.

**Figure 12:**
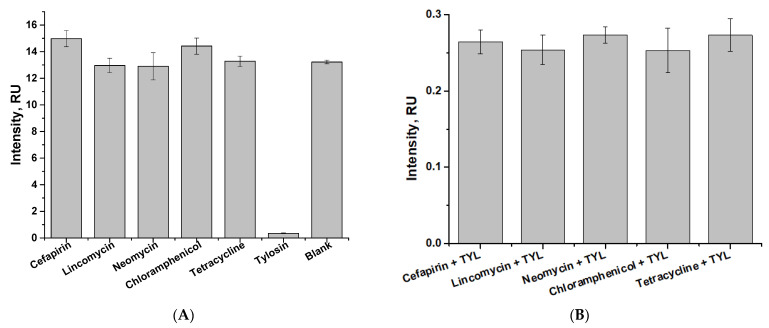
Selectivity of indirect LFIA for TYL. Dependencies of color intensity of the analytical zone on non-target analytes binding with specific antibodies (**A**), and tylosin binding with specific antibodies in the presence of non-target analytes (**B**).

According to the data obtained for non-target analytes (Figure 12A), the coloration intensity in the analytical zone corresponded to the values obtained in the absence of TYL (blank). At the same time, when TYL was added to the sample (100 ng/mL), there was no coloring of the analytical zone. As shown in Figure 12B, there was no interference in specific binding between tylosin and specific antibodies in the presence of other antibiotics (the color intensity of the analytical zone corresponded to the levels obtained with TYL binding, as shown in Figure 12A).

### 3.7. Comparison with Other Studies

To date, previously developed LFIAs for tylosin detection in various livestock products have LODs in the range of 0.09–31.25 ng/mL and 3–50 µg/kg, which correspond to the maximum residue limit of TYL in food of animal origin [37]. The developed LFIA with indirect labeling allows for a reduction in the detection limit of TYL by up to seven times with the same immunoreactants compared to LFIA with direct antibody labeling, which proves its effectiveness in comparison to other known LFIAs for tylosin (Table 7).

## 4. Conclusions

An LFIA based on direct labeling (where specific antibodies are conjugated with nanoparticles) and indirect labeling (where nanoparticles are conjugated with anti-species antibodies and specific antibodies are free in the solution) for the detection of tylosin in meat production has been described. According to the assessment of the analytical characteristics of the two LFIA formats, we can conclude that the main assay parameters affecting the LOD and color intensity of the analytical zone of the test strips are the concentrations of the reagents used, such as hapten–protein conjugates, specific antibodies, and the conjugate of GNP with antibodies. After choosing the optimal conditions to implement the analysis, it was noted that the LFIA with indirect antibody labeling provided an LOD up to seven times lower (visual: 1.6 μg/kg; instrumental: 0.14 μg/kg) compared to the LFIA with direct antibody labeling (visual: 8 μg/kg; instrumental: 1 μg/kg), which proves the effectiveness of this approach and allows for the determination of the target analyte in products of animal origin at low concentrations.

## Figures and Tables

**Table 1:** Changes in characteristics of tylosin LFIA with direct antibody labeling using varied incubation times of reagents.

Time of Reagent Incubation, min	Visual LOD, ng/mL	Instrumental LOD, ng/mL	Total Analysis Time, min	Coloration of the Analytical Zone, RU
0	4	0.37	5	11.0
1	4	0.77	6	11.8
3	4	0.94	8	11.4
5	4	0.34	10	11.1

**Table 2:** Changes in characteristics of tylosin LFIA with direct antibody labeling using varied Tween-20 concentrations in PBST.

Tween-20 Concentration, %	Visual LOD, ng/mL	Instrumental LOD, ng/mL	Total Assay Time, min	Coloration of the Analytical Zone, RU
0.2	4	1.03	10	8.9
0.5	4	0.48	7	9.1
1	4	0.37	5	11.0
2	4	0.46	5	10.9

**Table 3:** Changes in characteristics of tylosin LFIA with indirect antibody labeling for varied incubation times of reagents.

Time of Reagents Incubation, min	Visual LOD, ng/mL	Instrumental LOD, ng/mL	Total Assay Time, min	Coloration of the Analytical Zone, RU
0	0.8	0.04	10	6.7
1	0.8	0.01	11	10.2
3	0.8	0.08	13	10.1
5	0.8	0.03	15	14.6

**Table 4:** Changes in characteristics of tylosin LFIA with indirect antibody labeling for varied Tween-20 content in PBST.

Tween-20 Concentration, %	Visual LOD, ng/mL	Instrumental LOD, ng/mL	Total Assay Time, min	Coloration of the Analytical Zone, RU
0.2	0.2	0.05	15	3.6
0.5	0.8	0.03	15	5.2
1	0.8	0.03	15	14.6
2	0.8	0.04	15	11.7

**Table 5:** Characteristics of LFIA variants.

Number of Stages	Time of Reagents Incubation, min	Tween-20 Concentration, %	Visual LOD, ng/mL	Instrumental LOD, ng/mL	Total Assay Time, min	Coloration of Analytical Zone, RU
LFIA with direct antibody labeling						
1	0	1	4	0.4	5	11.0
LFIA with indirect antibody labeling						
1	5	1	0.8	0.03	15	14.6
LFIA with indirect antibody labeling and step-by-step addition of reagents						
2	-	1	4	0.03	10	5.7

**Table 6:** Recoveries of TYL in spiked meat samples for LFIA with indirect labeling (n = 3).

TYL Added, pg/mL	TYL Detected ± SD, pg/mL	Recovery, %
800	640 ± 21	80
160	122 ± 9	76
32	38 ± 3	119

**Table 7:** Characteristics of developed LFIAs for tylosin.

Label	Antibody Labeling	Limits of Detection		Reference
		ng per mL of Liquid Samples	mg per kg of Solid Samples	
GNP	Direct	Visual: 10 ng/mL (muscle, liver, fish and eggs)	-	[38]
Multi-residue fluorescent microsphere	Direct	Instrumental: 0.13 ng/mL (milk)	-	[39]
GNP	Direct	Visual: 10 ng/mL (honey)	-	[40]
GNP	Direct	Visual: 20 ng/mL (milk)	-	[41]
GNP	Direct	Visual: 8 ng/mL (milk)	Visual: 15 μg/kg (pork)	[42]
Latex microspheres	Direct	Visual: 4 ng/mL (milk)	Visual: 8 μg/kg (pork)	[42]
Time-resolved fluorescent microsphere	Direct	Visual: 2 ng/mL (milk)	Visual: 4 μg/kg (pork)	[42]
Amorphous carbon nanoparticles	Direct	Visual: 8 ng/mL (milk)	Instrumental: 11.44 μg/kg (chicken muscle); 15.96 μg/kg (bovine muscle); 22.29 μg/kg (porcine muscle); 25.53 μg/kg (porcine liver)	[24]
GNP	Direct	Instrumental: 1.72 ng/mL (milk, water)	-	[25]
Quantum dots	Direct	Instrumental: 1.84 ng/mL (milk) 2.69 ng/mL (honey)	-	[43]
Europium-chelated fluorescent microspheres	Direct	Visual: 31.25 ng/mL (milk) Instrumental: 1.72 ng/mL (milk)	-	[23]
GNP	Direct	-	Visual: 6 μg/kg (eggs)	[22]
Latex microspheres	Direct	-	Visual: 6 μg/kg (eggs)	[22]
Fluorescent microsphere	Direct		Visual: 3 μg/kg (eggs)	[22]
Fluorescent microspheres	Direct	Instrumental: 0.1 ng/mL (milk)	-	[44]
GNP	Direct	-	Instrumental: 0.05 mg/kg (milk)	[45]
GNP	Direct	Visual: 4 ng/mL (ground meat) Instrumental: 0.4 ng/mL (ground meat)	Visual: 8 μg/kg (ground meat) Instrumental: 1 μg/kg (ground meat)	This study
GNP	Indirect	Visual: 0.8 ng/mL (ground meat) Instrumental: 0.07 ng/mL (ground meat)	Visual: 1.6 μg/kg (ground meat) Instrumental: 0.14 μg/kg (ground meat)	This study

## Data Availability

The data presented in this study are available upon request from the corresponding author.

## Supplementary Materials

The following supporting information can be downloaded at https://www.mdpi.com/article/10.3390/s24216865/s1, Figure S1: Testing immunoreactants for TYL in ELISA. (a) Binding anti-TYL antibody with immobilized TYL–BSA conjugate. (b) Competitive detection of TYL, Table S1: Determination of the composition of GNP conjugates with antibodies, Table S2: Images of the test strips corresponding to the experimental results shown in Figure 5, Table S3: Images of the test strips corresponding to the experimental results shown in Table 1, Table S4: Images of the test strips corresponding to the experimental results shown in Table 2 and Figure 6, Table S5: Images of the test strips corresponding to the experimental results shown in Figure 8A, Table S6: Images of the test strips corresponding to the experimental results shown in Figure 8B, Table S7: Images of the test strips corresponding to the experimental results shown in Figure 9A, Table S8: Images of the test strips corresponding to the experimental results shown in Figure 9B, Table S9: Images of the test strips corresponding to the experimental results shown in Figure 11.

## References

[1] Manyi-Loh C., Mamphweli S., Meyer E., Okoh A. (2018). Antibiotic use in agriculture and its consequential resistance in environmental sources: Potential public health implications. Molecules.

[2] Tserenov I.V., Mosolov A.A., Slozenkina M.I., Buhtin A.A., Fedotova A.M., Mosolova N.I., Yuldashbaeva Z.Y. (2022). Impact of livestock production technology on ecology and environmental quality. IOP Conf. Ser. Earth Environ. Sci..

[3] Tiseo K., Huber L., Gilbert M., Robinson T.P., Van Boeckel T.P. (2020). Global trends in antimicrobial use in food animals from 2017 to 2030. Antibiotics.

[4] Ghimpețeanu O.M., Pogurschi E.N., Popa D.C., Dragomir N., Drăgotoiu T., Mihai O.D., Petcu C.D. (2022). Antibiotic use in livestock and residues in food-a public health threat: A review. Foods.

[5] Oliveira N., Gonçalves B., Lee S., Oliveira C., Corassin C. (2020). Use of antibiotics in animal production and its impact on human health. J. Food Chem. Nanotechnol..

[6] Robles-Jimenez L.E., Aranda-Aguirre E., Castelan-Ortega O.A., Shettino-Bermudez B.S., Ortiz-Salinas R., Miranda M., Li X., Angeles-Hernandez J.C., Vargas-Bello-Pérez E., Gonzalez-Ronquillo M. (2022). Worldwide traceability of antibiotic residues from livestock in wastewater and soil: A systematic review. Animals.

[7] Salam M.A., Al-Amin M.Y., Salam M.T., Pawar J.S., Akhter N., Rabaan A.A., Alqumber M.A.A. (2023). Antimicrobial resistance: A growing serious threat for global public health. Healthcare.

[8] Khmaissa M., Zouari-Mechichi H., Sciara G., Record E., Mechichi T. (2024). Pollution from livestock farming antibiotics an emerging environmental and human health concern: A review. J. Hazard. Mater. Adv..

[9] Chen J., Ying G.-G., Deng W.-J. (2019). Antibiotic residues in food: Extraction, analysis, and human health concerns. J. Agric. Food Chem..

[10] Nguyen B.-A.T., Chen Q.-L., He J.-Z., Hu H.-W. (2022). Livestock manure spiked with the antibiotic tylosin significantly altered soil protist functional groups. J. Hazard. Mater..

[11] Kivits T., Broers H.P., Beeltje H., van Vliet M., Griffioen J. (2018). Presence and fate of veterinary antibiotics in age-dated groundwater in areas with intensive livestock farming. Environ. Pollut..

[12] Trufanov D., Akimenko Y., Kolesnikov S., Kazeev K., Kumari A., Rajput V.D., Mandzhieva S.S., Minkina T., van Hullebusch E. (2024). Effects of veterinary antibiotics on the soil properties. Emerging Contaminants.

[13] Papich M.G., Papich M.G. (2016). Tylosin. Saunders Handbook of Veterinary Drugs.

[14] Aronson J.K., Aronson J.K. (2016). Tylosin. Meyler’s Side Effects of Drugs.

[15] Lin Q., de Waal T., Van Schepdael A., Adams E. (2019). A mass spectrometer-compatible liquid chromatographic method for the analysis of tylosin and its impurities using a superficially porous particle column. J. Pharm. Biomed. Anal..

[16] Granados-Chinchilla F., Sánchez J., García F., Rodríguez C. (2012). A novel green chemistry method for nonaqueous extraction and high-performance liquid chromatography detection of first-, second-, and third-generation tetracyclines, 4-epitetracycline, and tylosin in animal feeds. J. Agric. Food Chem..

[17] Huang J.-X., Yao C.-Y., Yang J.-Y., Li Z.-F., He F., Tian Y.-X., Wang H., Xu Z.-L., Shen Y.-D. (2019). Design of novel haptens and development of monoclonal antibody-based immunoassays for the simultaneous detection of tylosin and tilmicosin in milk and water samples. Biomolecules.

[18] Shan S., Lv X., Xiao X., Huang Y., Tong X., Lai W., Liu C., Wang S., Liu D. (2024). Integrated double signal amplification systems with ELISA assay for sensitive detection of tylosin in food. Food Agric. Immunol..

[19] Wei C., Wu A., Xu L., Xu C., Liu L., Kuang H., Xu X. (2023). Recent progress on lateral flow immunoassays in foodborne pathogen detection. Food Biosci..

[20] Wang P., Li J., Guo L., Li J., He F., Zhang H., Chi H. (2024). The developments on lateral flow immunochromatographic assay for food safety in recent 10 years: A review. Chemosensors.

[21] Di Nardo F., Chiarello M., Cavalera S., Baggiani C., Anfossi L. (2021). Ten Years of Lateral Flow Immunoassay Technique Applications: Trends, Challenges and Future Perspectives. Sensors.

[22] Liu Z., Chen J., Zhao S., Pang Y., Shen X., Lei H., Li X. (2021). Immunochromatographic assays based on three kinds of nanoparticles for the rapid and highly sensitive detection of tylosin and tilmicosin in eggs. Microchim. Acta.

[23] Wang Y., Yang J.-Y., He Y., Li L., Huang J.-X., Tian Y.-X., Wang H., Xu Z.-L., Shen Y.-D. (2021). Development of time-resolved fluorescence immunochromatographic assays for simultaneously detecting tylosin and tilmicosin in milk in group-screening manner. Foods.

[24] Song L., Wang Y., Li Q., Wang J., Gao T., An Z., Liu Q., Mao Y., Bu T., Huang X. (2023). Production of monoclonal antibody against tylosin and tilmicosin with homogeneous cross-reactivity and its application in lateral flow immunoassay. Microchim. Acta.

[25] Yang J., Wang Y., Zhang Y., Zeng X., Liu J., Tian Y., Wang H., Xu Z., Shen Y. (2023). Reverse distal similarity of hapten structure enhancing antibody’s group-specificity: Development of an immunochromatographic strip for tylosin and tilmicosin in milk and water. J. Food Compos. Anal..

[26] Frens G. (1973). Controlled nucleation for the regulation of the particle size in monodisperse gold suspensions. Nat. Phys. Sci..

[27] Sotnikov D.V., Barshevskaya L.V., Zherdev A.V., Dzantiev B.B. (2023). Enhanced Lateral Flow Immunoassay with Double Competition and Two Kinds of Nanoparticles Conjugates for Control of Insecticide Imidacloprid in Honey. Biosensors.

[28] Sotnikov D.V., Byzova N.A., Zherdev A.V., Dzantiev B.B. (2021). Retention of activity by antibodies immobilized on gold nanoparticles of different sizes: Fluorometric method of determination and comparative evaluation. Nanomaterials.

[29] Shim W.B., Kim J.S., Kim M.G., Chung D.H. (2013). Rapid and sensitive immunochromatographic strip for on-site detection of sulfamethazine in meats and eggs. J. Food Sci..

[30] Parolo C., Sena-Torralba A., Bergua J.F., Calucho E., Fuentes-Chust C., Hu L., Rivas L., Álvarez-Diduk R., Nguyen E.P., Cinti S. (2020). Tutorial: Design and fabrication of nanoparticle-based lateral-flow immunoassays. Nat. Protoc..

[31] Wang Z., Zhao J., Xu X., Guo L., Xu L., Sun M., Hu S., Kuang H., Xu C., Li A. (2022). An overview for the nanoparticles-based quantitative lateral flow assay. Small Methods.

[32] Thobhani S., Attree S., Boyd R., Kumarswami N., Noble J., Szymanski M., Porter R.A. (2010). Bioconjugation characterisation of gold colloid-labelled proteins. J. Immunol. Methods.

[33] Kaur K., Forrest J.A. (2012). Influence of particle size on the binding activity of proteins adsorbed onto gold nanoparticles. Langmuir.

[34] Davatgaran Taghipour Y., Kharrazi S., Amini S.M. (2018). Antibody Conjugated Gold. Nanoparticles for Detection of Small Amounts of Antigen. Based on Surface Plasmon. Reson. (SPR) Spectra. Nanomed. Res. J..

[35] Sotnikov D.V., Byzova N.A., Zherdev A.V., Dzantiev B.B. (2023). Ability of antibodies immobilized on gold nanoparticles to bind small antigen fluorescein. Int. J. Mol. Sci..

[36] Lei R., Wang D., Arain H., Mohan C. (2022). Design of gold nanoparticle vertical flow assays for point-of-care testing. Diagnostics.

[37] Commission Regulation (EU) 37/2010 of 22 December 2009 on Pharmacologically Active Substances and Their Classification Regarding Maximum Residue Limits in Foodstuffs of Animal Origin. https://eur-lex.europa.eu/legal-content/EN/TXT/PDF/?uri=CELEX:02010R0037-20240408.

[38] Le T., He H., Niu X., Chen Y., Xu J. (2013). Development of an immunochromatographic assay for detection of tylosin and tilmicosin in muscle, liver, fish and eggs. Food Agric. Immunol..

[39] Li X., Shen J., Wang Q., Gao S., Pei X., Jiang H., Wen K. (2015). Multi-residue fluorescent microspheres immunochromatographic assay for simultaneous determination of macrolides in raw milk. Anal. Bioanal. Chem..

[40] Song Y., Song S., Liu L., Kuang H., Guo L., Xu C. (2016). Simultaneous detection of tylosin and tilmicosin in honey using a novel immunoassay and immunochromatographic strip based on an innovative hapten. Food Agric. Immunol..

[41] Li X., Wen K., Chen Y., Wu X., Pei X., Wang Q., Liu A., Shen J. (2015). Multiplex immunogold chromatographic assay for simultaneous determination of macrolide antibiotics in raw milk. Food Anal. Methods.

[42] Li X., Wu X., Wang J., Hua Q., Wu J., Shen X., Sun Y., Lei H. (2019). Three lateral flow immunochromatographic assays based on different nanoparticle probes for on-site detection of tylosin and tilmicosin in milk and pork. Sens. Actuators B Chem..

[43] Lai X., Lv X., Zhang G., Xiong Z., Lai W., Peng J. (2020). Highly specific anti-tylosin monoclonal antibody and its application in the quantum dot bead-based immunochromatographic assay. Food Anal. Methods.

[44] Ashuo A., Zou W., Fu J., Yang T., Yu L., Liu W., Yang L., Mari G.M., Jiang H. (2020). High throughput detection of antibiotic residues in milk by time-resolved fluorescence immunochromatography based on QR code. Food Addit. Contam. Part A Chem. Anal. Control Expo. Risk Assess..

[45] Koike H., Hayashi M., Kazama K., Yoshikawa S., Hayashi H., Ohba Y., Matsushima Y., Nagano C., Kanda M., Otsuka K. (2023). Validation and application of an immunochromatographic test to detect four macrolides and two lincosamides in raw cow milk. Food Addit. Contam. Part A Chem. Anal. Control Expo. Risk Assess..

