# Supplementary material for: Comparative Characteristics of Immunochromatographic Test Systems for Tylosin Antibiotic in Meat Products
Source: Sensors (Basel). 2024 Oct 25;24(21):6865. doi: 10.3390/s24216865 (PMC11548405; doi:10.3390/s24216865)
Supplement: Supplementary file 1 [file sensors-24-06865-s001.zip › sensors-3267435-supplementary.pdf]

sensors

Supplementary Information

Comparative Characteristics of Immunochromatographic Test Systems for Tylosin Detection in Meat Products

Lyubov V. Barshevskaya, Dmitriy V. Sotnikov, Elena A. Zvereva, Boris B. Dzantiev * and Anatoly V. Zherdev

A.N. Bach Institute of Biochemistry, Research Center of Biotechnology of the Russian Academy of Sciences, Leninsky prospect 33, Moscow 119071, Russia
* Correspondence: dzantiev@inbi.ras.ru, Tel.: +7-495-954-31-42

Characterization of immunoreagents

Enzyme immunoassay was used to assess the reactivity of monoclonal antibodies to TYL. The study of their binding with the immobilized TYL–BSA conjugate is presented in Figure 1. The obtained dependence allowed for choosing the antibody concentration for competitive assay as the value providing OD_{450} = 1.0 and, in this way, good amplitude of competitive curve. Thus, we chose the antibody concentration equal to 55 ng/mL and realized the competitive ELISA with its use. As follows from Figure 1B, the limit of detection (LOD) in competitive ELISA of TYL was 1,4 ng/mL, and the operating range was 0.5–11 ng/mL. Thus, the immunoreagents are suitable for use in LFIA development.

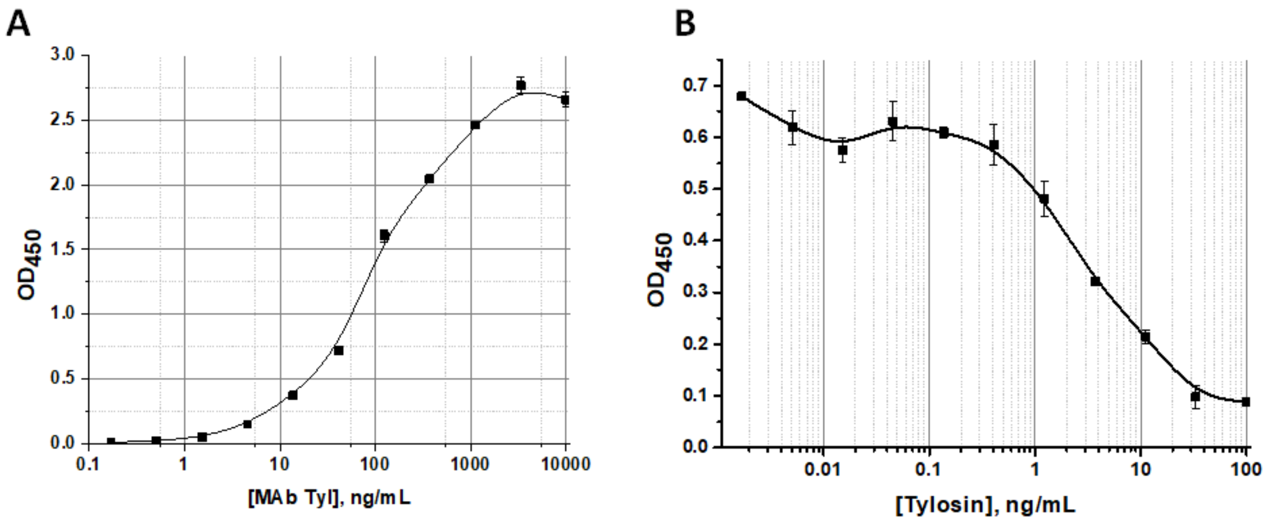

Figure S1. Testing immunoreactants for TYL in ELISA. (**A**) Binding anti-TYL antibody with immobilized TYL–BSA conjugate. (**B**) Competitive detection of TYL.

Full protocol and experimental data for determination of the antibodies amount conjugated with GNPs

The content of GNPs in the prepared colloidal solution was estimated basing on the concentration of the used $HAuCl_4$ and the TEM data indicating GNPs diameter equal to 24.5 nm. The given content was found to be $3.37 \cdot 10^{11}$ GNPs per mL. Note that the minimal area of GNP – IgG contact accords to transverse area for Fc region of IgG and by this way is equal to 19.6 nm^2. So monolayer coating of the surface of one nanoparticle (1885 nm^2) is possible with a maximum ratio of antibodies to nanoparticles equal to 96:1. To have some excess of antibodies for the conjugation, the IgG : GNP ratio in the reaction mixture was 120 : 1. For this purpose, the IgG were added for reaching their concentrations in final reaction mixture with GNP equal to 10 μg/mL, i.e. $4 \cdot 10^{13}$ molecules per mL.

To determine the antibodies amount conjugated with GNPs the listed below protocol was used that is based on our earlier developments [1,2] and actual modifications. GNP conjugates with anti-TYL antibodies or GAMI without adding BSA as a stabilizer (since BSA also contains tryptophan residues) were centrifuged and the supernatants were collected. The resulting supernatants were poured into two groups of wells of 96-well white microplates (300 µL per well, for each supernatant – three wells in group G1 and G2). IgG solutions and water were taken for their simultaneous additions to the supernatants-containing wells, namely:

1a) 10 µL of stock IgG solutions to the supernatants of the conjugates between GNPs and the same IgG to reach 10 µg/mL of the added IgG in the final solutions (G1 wells);

1b) 10 µL of water to another wells containing the same supernatants (Gr2 wells);

2a) new 10 µL portions of stock IgG solutions to the Gr1 wells;

2b) the next 10 µL of water to the Gr2 wells;

3a) new 10 µL portions of stock IgG solutions to the Gr1 wells;

3b) the next 10 µL of water to the Gr2 wells.

The fluorescence values at 280 nm as excitation wavelength and 350 nm as emission wavelength (being characteristic for tryptophan) were measured after additions: 1a) and 1b); 2a) and 2b); 3a) and 3b). The difference in fluorescence in solutions containing and not containing the added antibodies corresponded to fluorescence of 10, 20, and 30 µg/mL of the antibodies in the supernatant media.

The registered fluorescence values were used to calculate the concentrations of antibodies in the supernatants using the following formula

$$C_{sup} = C_{added} * F_{sup} / (F_{mix} – F_{sup}) ,$$

where

C_{sup} – concentration of antibodies in supernatant of the conjugation reaction mixture, µg/mL

C_{added} – concentration of antibodies added to the supernatant, µg/mL

F_{sup} – fluorescence of the supernatant, rel. un.;

F_{mix} – fluorescence of the supernatant with the addition of antibodies, rel. un.

The difference between the concentration of antibodies added to the reaction solution (10 µg/mL) and the concentration of antibodies in the supernatant (C_{sup}) gives the concentration of antibodies in the corresponding conjugate with GNPs (C_{conj}, µg/mL).

The results of determining the conjugates composition are given in Table S1.

Table S1. Determination of the composition of GNP conjugates with antibodies.

C_{added}, µg/mL	F_{sup}, rel. un.	F_{mix}, rel. un.	$F_{added-per-1}$, rel. un.*	C_{sup}, µg/mL	C_{conj}, µg/mL	Degree of binding, %**
		**GNP-Anti-TYL antibodies conjugate**				
10	164.8	1544.8	138.0	1.2	8.8	88
20	137.8	2556.8	121.0	1.1	8.9	89
30	132.2	3612.8	116.0	1.1	8.9	89
		**GNP-GAMI conjugate**				
10	948	5572.7	462.5	2.0	8.0	80
20	792	9658.8	443.3	1.8	8.2	82
30	810.7	13856.5	434.9	1.9	8.1	81

* Calculated fluorescence for 1 µg/mL of antibodies added to the supernatants, rel. un.; ** Degree of antibodies binding in the course of conjugation with GNPs, %.

Notes to the technique:

- Since tryptophan fluorescence strongly depends on the medium composition, the additions of IgG to supernatants of GNP conjugates is used in the technique instead of direct fluorescence measurements for IgG in pure solutions.

- The addition of 10 μL volumes is not a strong demand. Another small volumes (much less than the volume of the supernatant in the well) can be added to the supernatants.
- One calibrating concentration of IgG is sufficient to determine the concentration of antibodies in the conjugates. However, three concentrations ensure that the measurements were carried out in region of linearity between the IgG concentration and fluorescence response. The close values calculated for these three concentrations confirm the absence of error.

LFIA with direct antibody labeling

Table S2. Images of the test strips corresponding to the experimental results shown in Figure 5.

**Tylosin-BSA in the analytical zone, mg/mL**				
0.1	0.2	0.5	1	2
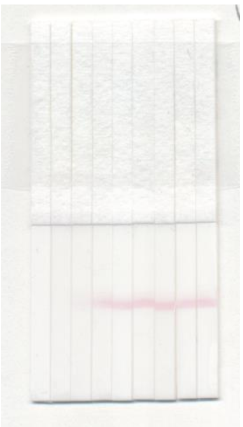		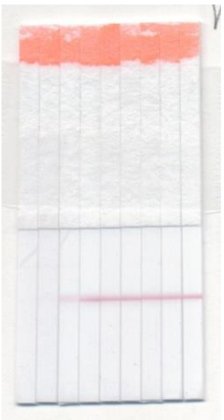	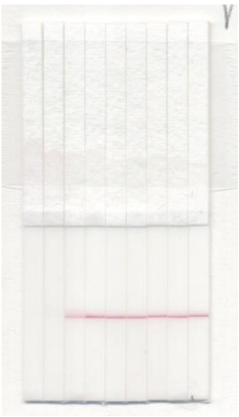	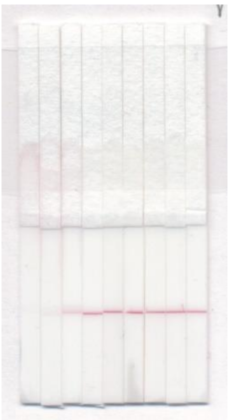
The concentrations of TYL in the samples for the test strips (from left to right) 100; 20; 4; 0.8; 0.2; 0.03; 0.006; 0.001; and 0.0 ng/mL, respectively.	The concentrations of TYL in the samples for the test strips (from left to right) 100; 20; 4; 0.8; 0.2; 0.03; 0.006; 0.001; and 0.0 ng/mL, respectively.	The concentrations of TYL in the samples for the test strips (from left to right) 100; 20; 4; 0.8; 0.2; 0.03; 0.006; 0.001; and 0.0 ng/mL, respectively.	The concentrations of TYL in the samples for the test strips (from left to right) 100; 20; 4; 0.8; 0.2; 0.03; 0.006; 0.001; and 0.0 ng/mL, respectively.	The concentrations of TYL in the samples for the test strips (from left to right) 100; 20; 4; 0.8; 0.2; 0.03; 0.006; 0.001; and 0.0 ng/mL, respectively.

Table S3. Images of the test strips corresponding to the experimental results shown in Table 1.

**Time of reagent incubation, min**			
0	1	3	5
	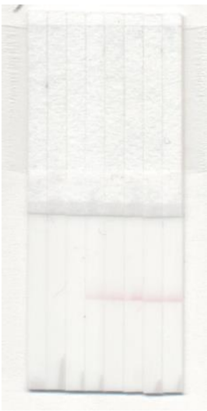	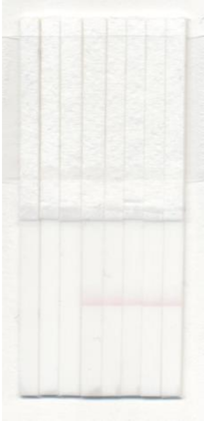	
The concentrations of TYL in the samples for the test strips (from left to right) 100; 20; 4; 0.8; 0.2; 0.03; 0.006 and 0.001 ng/mL, respectively.	The concentrations of TYL in the samples for the test strips (from left to right) 100; 20; 4; 0.8; 0.2; 0.03; 0.006 and 0.001 ng/mL, respectively.	The concentrations of TYL in the samples for the test strips (from left to right) 100; 20; 4; 0.8; 0.2; 0.03; 0.006 and 0.001 ng/mL, respectively.	The concentrations of TYL in the samples for the test strips (from left to right) 100; 20; 4; 0.8; 0.2; 0.03; 0.006 and 0.001 ng/mL, respectively.

Table S4. Images of the test strips corresponding to the experimental results shown in Table 2 and Figure 6.

**Tween-20 concentration, %**			
0.2	0.5	1	2
The concentrations of TYL in the samples for the test strips (from left to right) 100; 20; 4; 0.8; 0.2; 0.03; 0.006 and 0.001 ng/mL, respectively.	The concentrations of TYL in the samples for the test strips (from left to right) 100; 20; 4; 0.8; 0.2; 0.03; 0.006 and 0.001 ng/mL, respectively.	The concentrations of TYL in the samples for the test strips (from left to right) 100; 20; 4; 0.8; 0.2; 0.03; 0.006 and 0.001 ng/mL, respectively.	The concentrations of TYL in the samples for the test strips (from left to right) 100; 20; 4; 0.8; 0.2; 0.03; 0.006 and 0.001 ng/mL, respectively.

LFIA with indirect antibody labeling

Table S5. Images of the test strips corresponding to the experimental results shown in Figure 8a.

**Tylosin-BSA in the analytical zone, mg/mL**		
0.2	0.5	1
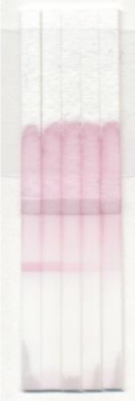	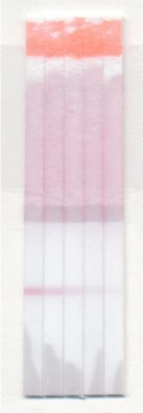	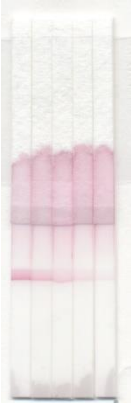
The concentrations of anti-TYL antibodies (from left to right) 100; 50; 25; 12.5 and 6.25 ng/mL, respectively.	The concentrations of anti-TYL antibodies (from left to right) 100; 50; 25; 12.5 and 6.25 ng/mL, respectively.	The concentrations of anti-TYL antibodies (from left to right) 100; 50; 25; 12.5 and 6.25 ng/mL, respectively.

Table S6. Images of the test strips corresponding to the experimental results shown in Figure 8b.

**Anti-TYL antibodies, ng/mL**		
12.5	25	50
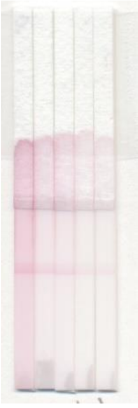	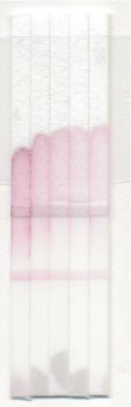	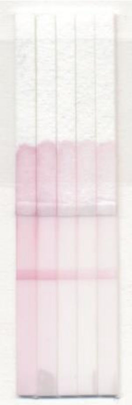
The concentrations of GNP-GAMI conjugate (from left to right) 2.0; 1.0; 0.5; 0.25 and 0.125 OD_{520}, respectively.	The concentrations of GNP-GAMI conjugate (from left to right) 2.0; 1.0; 0.5; 0.25 and 0.125 OD_{520}, respectively.	The concentrations of GNP-GAMI conjugate (from left to right) 2.0; 1.0; 0.5; 0.25 and 0.125 OD_{520}, respectively.

Table S7. Images of the test strips corresponding to the experimental results shown in Figure 9a.

**Time of reagent incubation, min**			
0	1	3	5
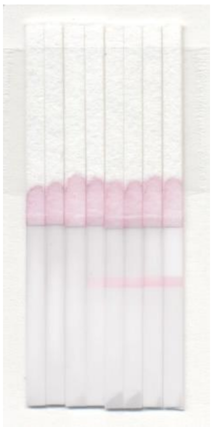	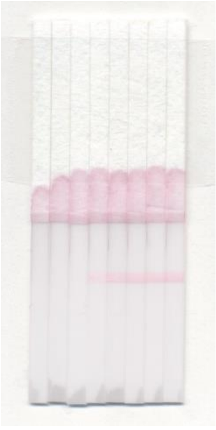	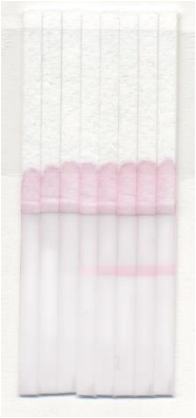	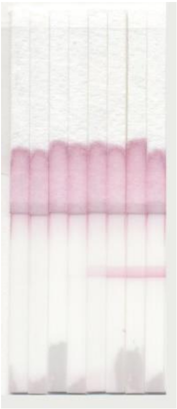
The concentrations of TYL in the samples for the test strips (from left to right) 100; 20; 4; 0.8; 0.2; 0.03; 0.006 and 0.001 ng/mL, respectively.	The concentrations of TYL in the samples for the test strips (from left to right) 100; 20; 4; 0.8; 0.2; 0.03; 0.006 and 0.001 ng/mL, respectively.	The concentrations of TYL in the samples for the test strips (from left to right) 100; 20; 4; 0.8; 0.2; 0.03; 0.006 and 0.001 ng/mL, respectively.	The concentrations of TYL in the samples for the test strips (from left to right) 100; 20; 4; 0.8; 0.2; 0.03; 0.006 and 0.001 ng/mL, respectively.

Table S8. Images of the test strips corresponding to the experimental results shown in Figure 9b.

**Tween-20 concentration, %**			
0.2	0.5	1	2
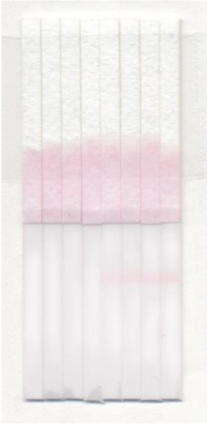	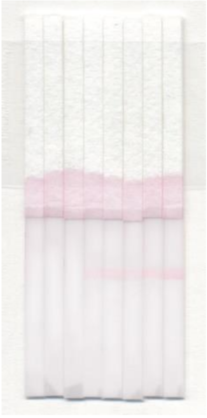	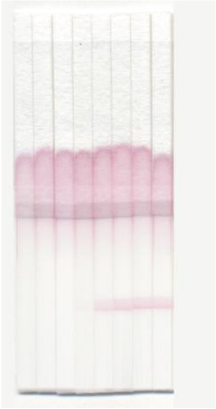	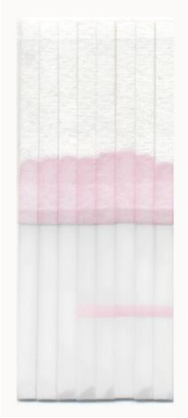
The concentrations of TYL in the samples for the test strips (from left to right) 100; 20; 4; 0.8; 0.2; 0.03; 0.006 and 0.001 ng/mL, respectively.	The concentrations of TYL in the samples for the test strips (from left to right) 100; 20; 4; 0.8; 0.2; 0.03; 0.006 and	The concentrations of TYL in the samples for the test strips (from left to right) 100; 20; 4; 0.8; 0.2; 0.03; 0.006 and	The concentrations of TYL in the samples for the test strips (from left to right) 100; 20; 4; 0.8; 0.2; 0.03; 0.006 and 0.001 ng/mL, respectively.

	0.001 ng/mL, respectively.	0.001 ng/mL, respectively.	

Table S9. Images of the test strips corresponding to the experimental results shown in Figure 11.

PBST	Meat extract
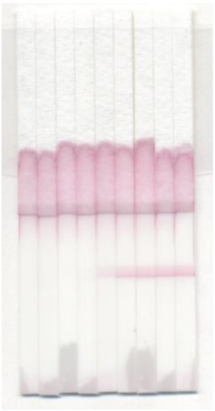	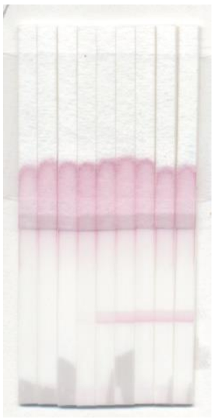
The concentrations of TYL in the samples for the test strips (from left to right) 100; 20; 4; 0.8; 0.2; 0.03; 0.006; 0.001; and 0.0 ng/mL, respectively.	The concentrations of TYL in the samples for the test strips (from left to right) 100; 20; 4; 0.8; 0.2; 0.03; 0.006; 0.001; and 0.0 ng/mL, respectively.

References

1. Sotnikov, D. V.; Byzova, N. A.; Zherdev, A. V.; Dzantiev, B. B. Retention of activity by antibodies immobilized on gold nanoparticles of different sizes: Fluorometric method of determination and comparative evaluation. *Nanomaterials* **2021**, *11* (11), 3117.https://doi.org/10.3390/nano11113117
2. Sotnikov, D.V.; Byzova, N.A.; Zherdev, A.V.; Dzantiev, B.B. Ability of antibodies immobilized on gold nanoparticles to bind small antigen fluorescein. *Int. J. Mol. Sci.* **2023,** 24 (23), 16967. https://doi.org/10.3390/ijms242316967
